# Self-extracellular RNA promotes pro-inflammatory response of astrocytes to exogenous and endogenous danger signals

**DOI:** 10.1186/s12974-021-02286-w

**Published:** 2021-11-02

**Authors:** Silvia Fischer, Emil Nasyrov, Monika Brosien, Klaus T. Preissner, Hugo H. Marti, Reiner Kunze

**Affiliations:** 1 Department of Biochemistry, Medical School, Justus-Liebig-University, Giessen, Germany; 2 Institute of Physiology and Pathophysiology, Department of Cardiovascular Physiology, Heidelberg University, Im Neuenheimer Feld 326, 69120 Heidelberg, Germany; 3 Department of Ophthalmology, University Eye Hospital, University of Tübingen, Tübingen, Germany; 4 German Center for Lung Research, Cardio-Pulmonary Institute, Universities of Giessen and Marburg Lung Center, Justus-Liebig-University, Giessen, Germany; 5 Department of Cardiology, Medical School, Kerckhoff-Heart-Research-Institute, Justus-Liebig-University, Giessen, Germany

**Keywords:** Extracellular RNA, Astrocyte, Inflammation, Stroke, Ischemia/reperfusion, PAMP, DAMP, TLR

## Abstract

**Objective:**

Astrocytes participate in the local innate immune response of the central nervous system. In response to stress such as ischemia, activated cells release endogenous factors known as damage-associated molecular patterns (DAMPs). Self-extracellular RNA (eRNA) is such a ubiquitous alarm signal. However, it is unclear whether eRNA is involved in the early acute phase of cerebral ischemia and is sufficient to sensitize astrocytes towards a DAMP or PAMP (pathogen-associated molecular pattern) reaction.

**Methods:**

Pro-inflammatory activation upon eRNA stimulation was characterized in primary murine astrocyte cultures. In vivo, an experimental stroke model was used to localize and quantify eRNA in murine brain sections. Using primary cortical neurons and the mouse hippocampal neuronal cell line HT-22, neuronal RNA release upon stress conditions related to cerebral hypoxia/ischemia was analyzed.

**Results:**

While low-dose eRNA alone did not promote pro-inflammatory activation of astrocytes in culture, it strongly enhanced the expression of pro-inflammatory cytokines in the presence of either Pam2CSK4, a synthetic PAMP molecule that mimics bacterial infection, or high mobility group box 1 (HMGB1), a prominent DAMP. Synergism of eRNA/Pam2CSK4 and eRNA/HMGB1 was prevented by blockage of the astroglial toll-like receptor (TLR)-2. Inhibition of NF-κB- and mitogen-activated protein kinase-dependent signaling pathways hampered eRNA/Pam2CSK4-mediated pro-inflammatory activation of astrocytes. In vivo, the amount of non-nuclear, presumably extracellular ribosomal RNA in close proximity to neurons significantly accumulated across the infarct core and peri-infarct areas that was accompanied by transcriptional up-regulation of various pro-inflammatory factors. Accordingly, the exposure of neurons to hypoxic/ischemic stress in vitro resulted in the release of eRNA, partly mediated by active cellular processes dependent on the cytosolic calcium level.

**Conclusion:**

The DAMP signal eRNA can sensitize astrocytes as active players in cerebral innate immunity towards exogenous and endogenous activators of inflammation (PAMPs and DAMPs) in a synergistic manner via TLR2-NF-κB-dependent signaling mechanisms. These findings provide new insights into the pathogenesis of ischemic stroke and other inflammatory neurological disorders. Further studies will clarify whether administration of RNase in vivo may serve as an effective treatment for inflammatory brain pathologies.

**Supplementary Information:**

The online version contains supplementary material available at 10.1186/s12974-021-02286-w.

## Background

In addition to their homeostatic function in the central nervous system (CNS), astrocytes, the most abundant glia cell type of the CNS, participate in the local innate immune response triggered by a variety of insults [1, 2]. Astrocytes respond to infection or injury not only by the release of cytokines and chemokines, but also participate in the removal of cell debris or microorganisms by phagocytosis [3].

The innate immune system protects against infections by the recognition of specific patterns or sequences/structures present on the surface of potential pathogens known as pathogen-associated molecular patterns (PAMPs) via pattern recognition receptors (PRRs) such as cell membrane or endosomal Toll-like receptors (TLRs). Activation of these PRRs initiates an inflammatory immune response through the intracellular mobilization of nuclear factor-kappa B (NF-κB) and other transcription factors to induce the release of cytokines and chemokines in immune and non-immune cells. As a consequence, the inflammatory environment is sensitized to help removing pathogens or damaging factors and to restore tissue homeostasis [4].

In response to stress conditions such as ischemia/reperfusion (I/R) injury or mechanical trauma, activated or damaged cells release endogenous factors that can also bind to PRRs and cause a harmful aseptic inflammatory response known as “sterile inflammation”. These endogenous factors are designated as damage-associated molecular patterns (DAMPs) or alarmins [5]. DAMPs include cytosolic, mitochondrial, or nuclear molecules such as heat shock proteins, histones, hyaluronan, uric acid crystals, thioredoxin, adenosine triphosphate, high mobility group box protein 1 (HMGB1), cold-inducible RNA-binding protein, as well as numerous self-nucleic acids (including nuclear DNA, messenger RNA, ribosomal RNA, microRNA) [6].

During the previous decade, self-extracellular RNA (eRNA, predominantly consisting of rRNA) released under pathological conditions from damaged tissue or injured cells was identified as a new alarmin, which contributes to disease progression in ischemic stroke, thrombosis, myocardial infarction, atherosclerosis, rheumatoid arthritis and cancer [7–13]. Besides its multifunctional and damaging repertoire, eRNA was previously described to serve as a potent adjuvant, particularly for TLR2 ligands, to enhance their pro-inflammatory potential in a synergistic manner in macrophages [14].

Ischemic stroke results in neuronal cell death and the release of DAMPs that orchestrate innate and adaptive immune responses in the injured regions of the brain, which is characterized by glial activation, recruitment of peripheral immune cells, and the release of cytokines and chemokines [15]. Focal brain inflammation aggravates secondary brain injury by exacerbating blood–brain barrier damage, microvascular failure, brain edema, oxidative stress, and by directly inducing neuronal cell death [15].

Although astrocytes, in addition to microglia, are the primary cells that mediate innate immune responses in the CNS [2], it remains unclear whether eRNA is sufficient to sensitize astrocytes towards exogenous and endogenous activators of inflammation (PAMPs and DAMPs). Using primary brain cell cultures in vitro and a mouse model of focal ischemic stroke in vivo, we characterized the pro-inflammatory activation upon eRNA stimulation in astrocytes, investigated eRNA release in the early acute phase upon cerebral I/R injury, and finally analyzed the molecular mechanisms potentially leading to neuronal eRNA release during cerebral ischemia.

## Methods

### Experimental stroke model

Animal experiments, which were performed with 8-week-old female and male C57BL/6 mice, were approved by the local animal welfare committee (Regierungspräsidium Karlsruhe, Germany, permission number: 35-9185.81/G-45/18), conformed to the Guide for the Care and Use of Laboratory Animals published by the US National Institutes of Health, and were carried out in accordance with the recently published Animal Research: Reporting In Vivo Experiments (ARRIVE) guidelines (https://www.nc3rs.org.uk/arrive-guidelines). All mice were housed at constant room temperature (22 ± 2 °C) and relative humidity (50–55%) on a controlled 12:12 h light–dark cycle, and were provided with standard laboratory chow (LASQCdiet Rod16; LASvendi, Soest, Germany) and water ad libitum. All mice were randomly allocated to experimental groups. Investigators were blinded for animal treatment in all experiments and analyses. Evaluation of all read-out parameters was done independently and in a blinded fashion. Mice were anesthetized by a mixture of 2% isoflurane in, 70% N_2_O and remainder O_2_, and were maintained by reducing the isoflurane concentration to 1.0–1.5%. To induce focal cerebral ischemia, a 7–0 silicon rubber-coated nylon monofilament (Doccol Corporation, Redlands, USA) was introduced into the left internal carotid artery and pushed towards the left middle cerebral artery (MCA) as previously described [16]. The intraluminal suture was left for 60 min. Subsequently, animals were re-anesthetized and the occluding monofilament was withdrawn to allow reperfusion for 2, 4 or 12 h. For sham surgery, the mice underwent the same procedure without vessel occlusion. The animals were maintained at 37 °C during and after surgery until they were fully recovered from anesthesia. Then, mice were returned to their solitary cages in a heated (30 °C) environment with free access to food and water for the remaining time.

### Immunofluorescence staining, image acquisition and analysis

Mouse brains were removed and embedded into Tissue-Tek (Sakura Finetek, Staufen, Germany), and coronal tissue sections (10 µm thickness; + 0.5 mm relative to Bregma) were prepared using a Leica CM1520 cryostat (Leica Biosystems, Wetzlar, Germany) at a constant temperature of − 15 °C. Coronal brain cryosections were fixed with 4% paraformaldehyde in PBS for 20 min. followed by 15 min incubation in blocking solution consisting of 10% goat serum (Dianova, Hamburg, Germany) in PBS. Then, slices were incubated with primary antibodies against rRNA (ribosomal RNA; 1:200; Novus Biologicals, Cambridge, UK, #NB100-662) and NeuN (neuronal nuclei; 1:500; Cell Signaling Technology, Frankfurt am Main, Germany, #12943) overnight at 4 °C followed by incubation with Cy3- and Cy2-conjugated anti-IgG antibodies (1:200; Dianova, #115-165-003, #111-225-003) for 1 h. All antibodies were diluted in LowCross-Buffer (Candor Bioscience, Wangen, Germany #100125). Slices were incubated for 10 min with 0.02% DAPI (Thermo Fisher Scientific, Darmstadt, Germany, #D1306) in PBS to stain nuclei followed by embedding in Mowiol mounting medium (Polysciences, Hirschberg an der Bergstrasse, Germany, #17951). Stained brain sections were scanned with 20-fold magnification using a Plan-Apochromat 20x/0.8 objective (Carl Zeiss Microscopy, Göttingen, Germany) mounted on a Zeiss Axiovert 200 microscope (Carl Zeiss Microscopy). Whole-section image acquisition was performed with an Orca Flash 4.0 camera (Hamamatsu Photonics, Herrsching am Amersee, Germany) and captured images were processed by TissueFAXS slides 4.2 software (TissueGnostics, Vienna, Austria) into TIFF files. Acquired images were analyzed using FIJI software (National Institutes of Health, Bethesda, MD, USA). Advanced macros were designed and optimized for automated, user-independent signal segmentation, co-localization and counting. The ipsi- and contralateral hemisphere, striatum and cortex were identified as regions of interest (ROIs) according to the Allen mouse brain atlas (https://mouse.brain-map.org/static/atlas). First, the space between DAPI^+^ nuclei was divided by equidistant borders into individual compartments associated to each nucleus. These individual compartments were further divided by a peri-nuclear ring (radius of + 5 µm in addition to each nucleus) into peri-nuclear and non-nuclear sections, which surrogate cytoplasmic and extracellular compartments, respectively. Next, nuclei and their associated peri- and non-nuclear compartments were categorized as either neuronal (NeuN^+^/DAPI^+^ nuclei) or non-neuronal (NeuN^−^/DAPI^+^ nuclei). The area of rRNA^+^ signal was measured separately in each of these compartments for neuronal and non-neuronal cells in each anatomical region of interest. Analyzed areas were further divided by the number of regional neuronal and non-neuronal nuclei, respectively, to reveal the cell-specific abundance of nuclear, peri- and non-nuclear rRNA in µm^2^ per cell category. The results represent mean values of two adjacent tissue sections analyzed per animal.

### Cell culture

Primary mixed glial cell cultures were prepared from brains of newborn C57BL/6 mice (P0–2) as described previously [17]. After 5 days, residual microglial cells were eliminated by treatment with 50 mM l-leucine methyl ester (Sigma-Aldrich, Steinheim, Germany, #L1002) for 1 h, and microglia-free astrocytes were cultured for further 7–9 days. Then, cells were harvested, seeded at 20,000 cells/cm^2^ in DMEM (Thermo Fisher Scientific, #41966,29) supplemented with 100 units/ml penicillin, 100 μg/ml streptomycin and 10% FBS, and were cultured upon reaching confluence. Cells were cultured for 4 h in serum-free medium in the absence or presence of either Bay 11-7084 (Cayman Chemical, Ann Arbor, MI, USA, #10010266; 5 µM), MAb-mTLR2 (InvivoGen, Toulouse, France, #mab2-mtlr2; 1 µg/ml), PD98059 (Merck Millipore, Darmstadt, Germany, #513000; 20 µM), or SB203580 (Merck Millipore, #559395; 10 µM). Subsequently, cells were treated in serum-free medium for 8 h with Pam2CSK4/eRNA molecule complexes, prepared by pre-incubation of Pam2CSK4 (InvivoGen, # tlrl-pm2s-1) and eRNA at indicated concentrations in PBS for 2 h at 37 °C. Alternatively, cells were treated in serum-free medium for 8 h with 10–500 ng/ml rhHMGB1 (R&D Systems, Wiesbaden, Germany, #1690-HMB) in the presence or absence of 1 µg/ml eRNA.

Cortical neurons from neonatal C57BL/6 mice (P0–1) were prepared and maintained as described previously [18]. After 8 days in vitro (DIV) growth medium was replaced with defined medium consisting of a mixture of a buffered saline solution (10 mM HEPES, pH 7.4, 114 mM NaCl, 26.1 mM NaHCO_3_, 5.3 mM KCl, 1 mM MgCl_2_, 2 mM CaCl_2_, 5 mM glucose, 1 mM glycine, 0.5 mM C_3_H_3_NaO_3_, and 0.001% phenol red) and phosphate-free Eagle’s minimum essential medium (9:1 v/v) supplemented with insulin (7.5 μg/ml), transferrin (7.5 μg/ml), and sodium selenite (7.5 ng/ml) (ITS supplement; Sigma-Aldrich, #1884-1VL). DIV10 neurons were treated with 10 µM glycine and either 10 or 20 µM N-methyl-D-aspartic acid (NMDA; Sigma-Aldrich, #M3262) for 1 or 2 h.

The mouse hippocampal neuronal cell line HT-22 [19] was seeded at 20,000 cells/cm^2^ in DMEM (Thermo Fisher Scientific, #41966029) containing 100 units/ml penicillin, 100 μg/ml streptomycin and 10% FBS. Upon reaching 80% confluence, neuronal cells were cultured in either deoxygenated phenol-free high glucose DMEM (Thermo Fisher Scientific, #21063029) or glucose-free DMEM (Thermo Fisher Scientific, #A1443001) supplemented with 100 units/ml penicillin, 100 μg/ml streptomycin and 1% FBS in an Invivo2 Plus hypoxia workstation (Ruskinn, Leeds, UK) flooded with humidified gas mixture consisting of 5% CO_2_ and ~ 94% N_2_ (1% O_2_) at 37 °C for 1, 2 or 4 h.

### Calcium imaging

The intracellular Ca^2+^ concentration was measured using Fura-2 AM (Thermo Fisher Scientific, #F1221; 5 µM) solved in Hepes ringer solution (HR) containing 136.4 mM NaCl, 5.6 mM KCl, 1 mM MgCl_2_ * 6 H_2_O, 2.2 mM Ca_2_Cl * H_2_O and 11 mM glucose. HT-22 cells were seeded at 7 × 10^4^ cells/cm^2^ on glass bottom µ-dishes with two well silicone inserts (Ibidi, Martinsried, Germany, #81176). Upon reaching sub-confluence, cells were differentiated in Neurobasal medium (Thermo Fisher Scientific, #21103049) containing 2 mmol/l glutamine, 1 × N2 supplement (Thermo Fisher Scientific, #17502048), 50 ng/ml nerve growth factor-β (Thermo Fisher Scientific, #A42627), 100 µM phorbol-12,13-dibutyrate (Sigma-Aldrich, #524390), 100 µM dibutyryl cAMP (Santa Cruz Biotechnology, Heidelberg, Germany, #sc-201567) for 48 h. Then, cells were washed with HR and loaded with fura2-AM for 30 min. at 37 °C, 5% CO_2_ and 21% O_2_. After loading, silicone chambers were removed and the baseline fluorescence intensity induced by 340 nm and 380 nm excitation and emitted at 510 nm was measured under normoxic conditions using a fluorescence microscope (DMI 6000, Leica Microsystems, Wetzlar, Germany). Normoxic or hypoxic HR was applied after 2 min, and changes in fluorescence intensity were recorded. Cells showing instable/quenched fluorescence signals were excluded from analysis. The fluorescence intensity ratio between 340 and 380 nm excitation wavelength, which is directly proportional to the free Ca^2+^ concentration, was calculated. Values are shown as F1/F0 (F1, Fura-2 340/380 ratio at indicated time point; F0, Fura-2 340/380 ratio at baseline).

### Isolation and quantification of eRNA

RNA used to stimulate cells was purified from confluent cultures of fibroblasts using TRIzol reagent (Thermo Fisher Scientific, #15596026) according to manufacturer's instructions. eRNA of cell supernatants was isolated using an extraction kit from Biozym (Hessisch Oldendorf, Germany) and quantified using Quant-iT RNA Assay Kit (Thermo Fisher Scientific, #Q33140).

### RNase activity assay

RNase activity was determined according to the method described by [20] with some minor modifications. Briefly, 100 μl of supernatant or cell lysate were added to 50 μl of RNase buffer (50 mM Tris–HCl, 130 mM NaCl, 2 mM EDTA, and 0.1 mg/ml acetylated-BSA, pH 8.0) and 100 μl of poly:C (400 μg/ml) followed by 5 min incubation at 37 °C. One hundred μl aliquots were mixed with 250 μl of ice-cold 6% perchloric acid and 20 mM lanthanum chloride. Then, 100 μl of a 10 mg/ml fatty acid-free-BSA solution were added and mixtures were maintained on ice for 15 min and centrifuged for 15 min at 16,000*g* at 4 °C. Substrate degradation was determined by measuring the absorbance of the supernatant at 280 nm. All activity values were normalized to the same cell number.

### Analysis of cell viability

Cellular viability was determined using the LDH cytotoxicity assay (Thermo Fisher Scientific, #88953) according to manufacturer's instructions.

### Enzyme-linked Immunosorbent Assay (ELISA)

IL-6 and TNF-α protein levels in cell supernatants were measured by quantitative ELISA (Thermo Fisher Scientific, #88-7324-86, #88-7064-86) according to manufacturer's instructions.

### Quantitative real-time RT-PCR analysis

RNA isolation from tissue samples or cells, cDNA synthesis, and quantitative real-time PCR were performed as described recently [21]. Primer sequences are listed in Table 1.

**Table 1:** List of primers used for quantitative real-time RT-PCR

Gene	Forward primer sequence (5′–3′)	Reverse primer sequence (5′–3′)
*Il-6*	AGTTGCCTTCTTGGGACTGA	TCCACGATTTCCCAGAGAAC
*Mcp-1*	CCCAATGAGTAGGCTGGAGA	TCTGGACCCATTCCTTCTTG
*Rps12*	GAAGCTGCCAAAGCCTTAGA	AACTGCAACCAACCACCTTC
*Tlr1*	CTTACCAGAGTGCCCAAGGA	ACCCTCAGCTTGGACAATGA
*Tlr2*	CTGAGAATGATGTGGGCGTG	TTAAAGGGCGGGTCAGAGTT
*Tlr3*	TTGCGTTGCGAAGTGAAGAA	TGTTCAAGAGGAGGGCGAAT
*Tlr4*	TGGGTGAGAAATGAGCTGGT	ACCACAATAACCTTCCGGCT
*Tnf*	CGTCAGCCGATTTGCTATCT	CGGACTCCGCAAAGTCTAAG

*Il-6* interleukin-6, *Mcp-1* monocyte chemoattractant protein-1, *Rps12* ribosomal protein S12, *Tlr* Toll-like receptor, *Tnf* tumor necrosis factor alpha

### Statistical analysis

If not indicated otherwise, all results are expressed as single values and mean ± SD. Differences between two independent experimental groups were analyzed by two-tailed Student's *t* test. Differences of one parameter among three or more independent experimental groups were analyzed using one-way ANOVA followed by a Holm–Sidak’s multiple comparisons test. Differences of two parameters among two or more independent experimental groups were analyzed by two-way ANOVA followed by a Holm–Sidak’s multiple comparisons test. A probability value of *p* < 0.05 was considered statistically significant. Data plotting and statistical analyses were done with Prism 6 (GraphPad Software, La Jolla, CA, USA).

## Results

### Self-extracellular RNA-PAMP-TLR signaling promotes pro-inflammatory gene expression in astrocytes.

**Fig. 1:**
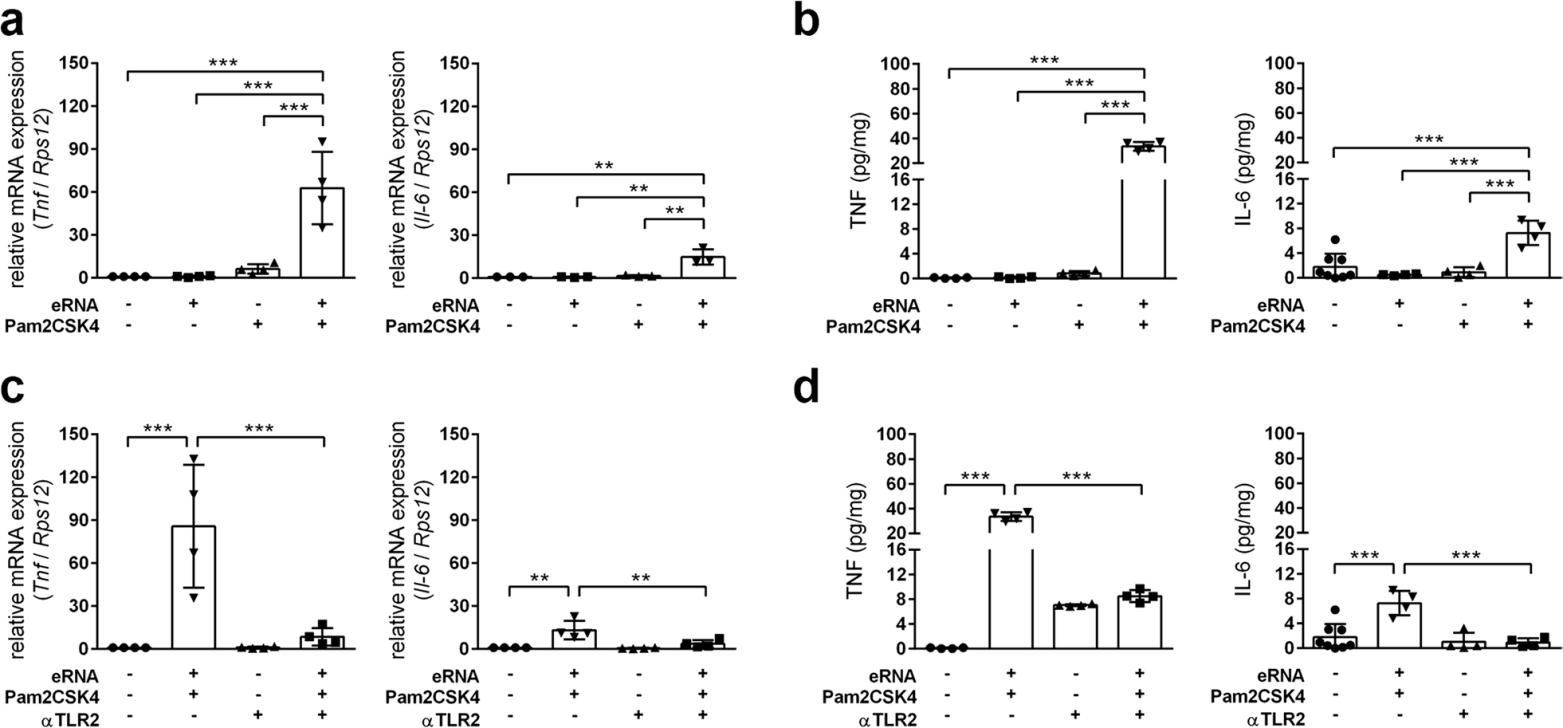
Self-extracellular RNA/Pam2CSK4 complex promotes pro-inflammatory gene expression in astrocytes through TLR2.** a**, **b** Primary astrocytes isolated from brains of neonatal mice were treated for 8 h with either 100 pg/ml Pam2CSK4, 1 µg/ml eRNA or Pam2CSK4/eRNA molecule complexes, prepared by co-incubation of 100 pg/µl Pam2CSK4 and 1 µg/µl RNA for 2 h at 37 °C. **c**, **d** Astrocytes were treated for 4 h with 1 µg/ml MAb-mTLR2 prior to stimulation with Pam2CSK4/eRNA molecule complexes for 8 h. Untreated cells served as control. **a**, **c** Real-time RT-PCR was used to determine transcript levels of pro-inflammatory cytokines. Values are normalized to *Rps12* (*n* = 3–4 per group; One-way ANOVA with Holm–Sidak's multiple comparisons test; * *p* < 0.05, ** *p* < 0.01, *** *p* < 0.001). **b,** **d** TNF-α and IL-6 protein levels in cellular supernatants were quantified by ELISA (*n* = 3–4 per group; One-way ANOVA with Holm–Sidak's multiple comparisons test; * *p* < 0.05, ** *p* < 0.01, *** *p* < 0.001)

We first analyzed whether eRNA reinforces the pro-inflammatory properties of Pam2CSK4, a synthetic molecule that mimics bacterial infection. Treatment of primary murine astrocytes with preformed complexes of low-dose eRNA and Pam2CSK4 significantly increased transcript levels of pro-inflammatory cytokines including *Tnf* and *Il-6*, whereas eRNA and Pam2CSK4 alone did not affect the pro-inflammatory gene expression (Fig. 1a). Accordingly, the release of TNF-α and IL-6 protein was increased by eRNA/Pam2CSK4 complexes (Fig. 1b). Upon treatment with eRNA/Pam2CSK4 complexes, astrocytes exhibited increased *Il-1b* mRNA levels, whereas the extracellular level of IL-1β protein was not altered (data not shown). The pre-treatment of eRNA with RNase almost completely blunted the pro-inflammatory response of astrocytes to eRNA/Pam2CSK4 complexes (Additional file 1: Fig. S1).

Pam2CSK4 is a potent agonist for TLR2/TLR6 heterodimers. Accordingly, blockage of astroglial TLR2 using neutralizing antibodies significantly suppressed up-regulation of TNF-α and IL-6 expression upon eRNA/Pam2CSK4 stimulation (Fig. 1c, d).

**Fig. 2:**
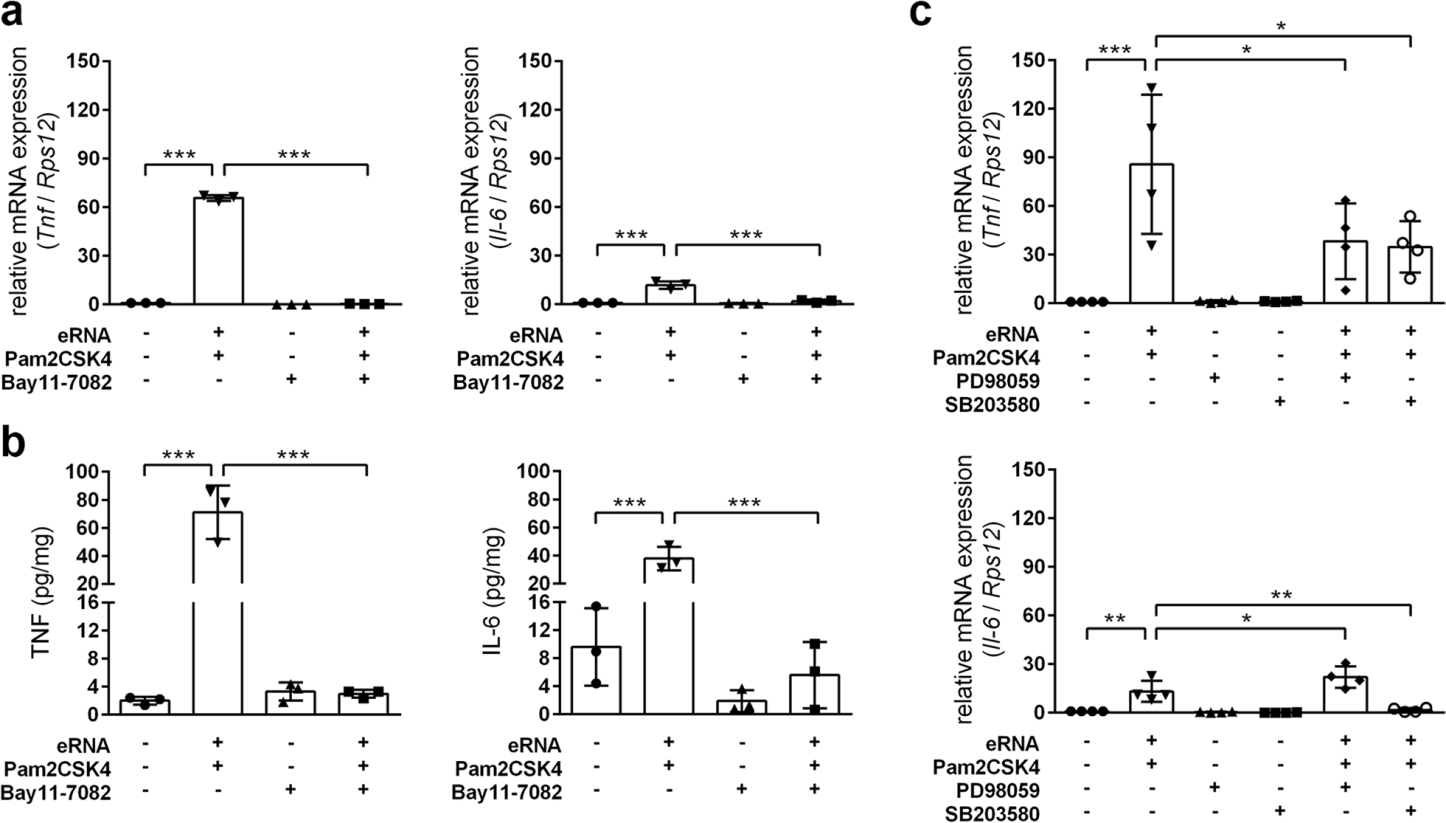
Self-extracellular RNA/Pam2CSK4 complex promotes pro-inflammatory gene expression in astrocytes in an NF-κB- and MAPK-dependent manner. Astrocytes were treated for 4 h with either (**a**, **b**) 5 µM Bay 11–7084, **c** 20 µM PD98059 or 10 µM SB203580 prior to 8 h stimulation with Pam2CSK4/eRNA molecule complexes, prepared by co-incubation of 100 pg/µl Pam2CSK4 and 1 µg/µl RNA for 2 h at 37 °C. Untreated cells were used as control. **a**, **c** Real-time RT-PCR was used to determine transcript levels of pro-inflammatory cytokines. Values are normalized to *Rps12* (*n* = 3–4 per group; One-way ANOVA with Holm–Sidak's multiple comparisons test; * *p* < 0.05, ** *p* < 0.01, *** *p* < 0.001). **b** TNF-α and IL-6 protein levels in cellular supernatants were quantified by ELISA (*n* = 3–4 per group; One-way ANOVA with Holm–Sidak's multiple comparisons test; * *p* < 0.05, ** *p* < 0.01, *** *p* < 0.001)

Pre-treatment with BAY 11–7082, a potent inhibitor of the IκB kinase (key upstream regulator of NF-κB), completely prevented the eRNA/Pam2CSK4-induced pro-inflammatory activation of astrocytes (Fig. 2a, b). Moreover, pharmacological inhibition of mitogen-activated protein kinase kinase 1 and 2 (MAP2K1/2) using PD98059 prior to eRNA/Pam2CSK4 stimulation prevented transcriptional up-regulation of *Tnf*, but further increased *Il-6* expression (Fig. 2c). Pharmacological targeting of p38 MAPK using SB203580 potently blocked pro-inflammatory gene expression evoked through eRNA/Pam2CSK4 complexes (Fig. 2c). Further analyses revealed the up-regulation of *Tlr1-3* transcript levels by eRNA/Pam2CSK4 complexes in an NF-κB- and MAPK-dependent manner (Additional file 2: Fig. S2a, c, d). Only the up-regulation of *Tlr2* was prevented by TLR2 blockage (Additional file 2: Fig. S2b).

### Self-extracellular RNA and HMGB1 cooperatively promote pro-inflammatory activation of astrocytes.

**Fig. 3:**
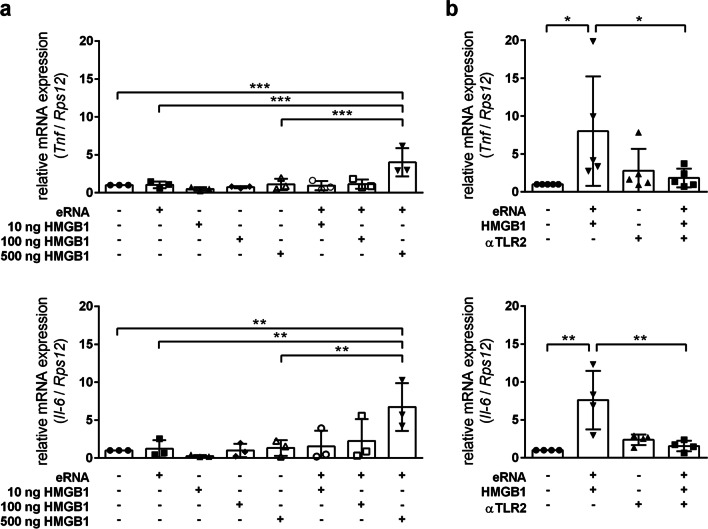
Self-extracellular RNA and HMGB1 cooperate to promote pro-inflammatory activation of astrocytes.** a** Astrocytes were treated for 8 h with 10–500 ng/ml rhHMGB1 in the presence or absence of 1 µg/ml eRNA. **b** Astrocytes were treated for 8 h with 1 µg/ml eRNA and 500 ng/ml rhHMGB1 in the presence or absence of 1 µg/ml MAb-mTLR2. Untreated cells were used as control. Real-time RT-PCR was used to determine transcript levels of pro-inflammatory cytokines. Values are normalized to *Rps12* (*n* = 3–5 per group; One-way ANOVA with Holm–Sidak's multiple comparisons test; * *p* < 0.05, ** *p* < 0.01, *** *p* < 0.001)

As mentioned above, DAMPs are released by cells under stress conditions and promote inflammation. A constantly growing number of DAMPs have been identified as endogenous ligands of TLRs. Among them HMGB1, an endogenous ligand of TLR2 and 4, is the most studied member and mediates inflammatory and immune reactions in the CNS. Thus, we analyzed whether eRNA and HMGB1 cooperatively promote inflammatory responses in astrocytes. Astrocytes co-treated with low-dose eRNA and 500 ng/ml HMGB1 exhibited significantly increased transcript levels of *Tnf*, and *Il-6*, whereas HMGB1 alone did not cause an enhanced pro-inflammatory cytokine expression (Fig. 3a). Up-regulation of *Tnf* and *Il-6* transcript levels by eRNA/HMGB1 co-stimulation was prevented upon blockage of TLR2 function (Fig. 3b). Moreover, among the TLRs that potentially bind eRNA or HMGB1, *Tlr2* expression was significantly elevated by eRNA/HMGB1 in a TLR2-dependent manner (Additional file 3: Fig. S3).

### Hypoxia/ischemia and cytosolic calcium rises trigger neuronal RNA release

Sterile neuroinflammation is a result of excessive liberation of DAMPs/alarmins by damaged nerve and glial cells undergoing necrosis or apoptosis, and occurs following acute brain insults such as stroke, hemorrhage, or trauma [22]. Thus, we investigated whether acute ischemic stroke might cause the release of rRNA, the predominant eRNA species, acting as DAMP in the immune response towards sterile inflammation [22]. We used co-immunofluorescence staining to evaluate the abundance of rRNA in nuclear, peri-nuclear (cytoplasm) and non-nuclear (extracellular space) compartments of neurons (NeuN^+^) and non-neuronal (NeuN^−^) cells. Increasing amounts of non-nuclear rRNA were assessed as a surrogate marker for cellular release of rRNA.

**Fig. 4:**
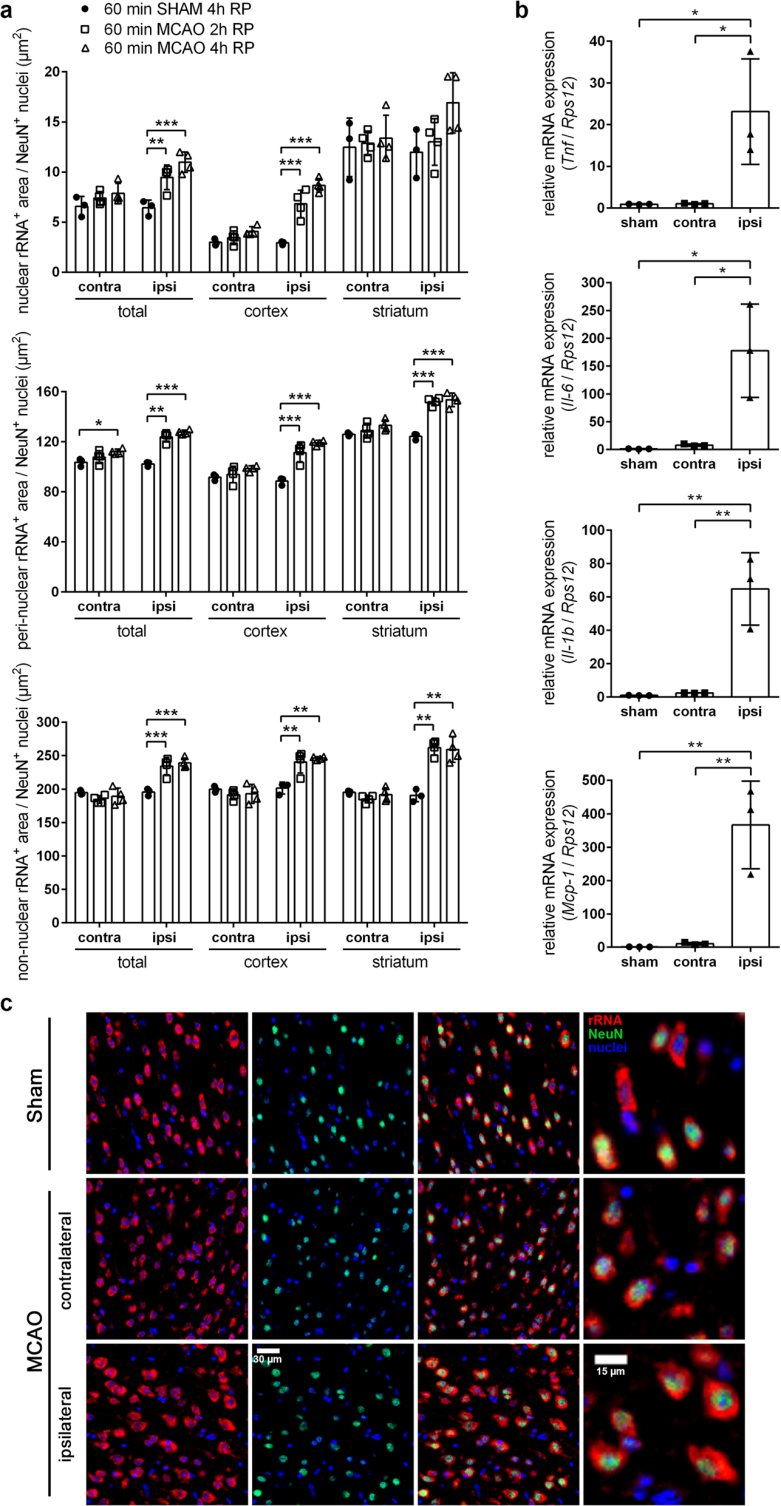
Acute cerebral I/R injury increases the non-nuclear abundance of neuronal-derived rRNA and promotes pro-inflammatory cytokine expression.** a** Mice underwent 60 min MCAO followed by 2 h or 4 h of reperfusion or were subjected to sham surgery. Co-immunofluorescent staining was performed to determine the nuclear, peri-nuclear and non-nuclear abundance of neuronal rRNA across the striatum and cortex of the contra- and ipsilateral brain hemisphere (*n* = 3–4 per group; One-way ANOVA with Holm–Sidak's multiple comparisons test; * *p* < 0.05, ** *p* < 0.01, *** *p* < 0.001). **b** Mice underwent 60 min MCAO followed by 12 h reperfusion or were subjected to sham surgery. Real-time RT-PCR was used to determine transcript levels of pro-inflammatory cytokines. Values are normalized to *Rps12* (*n* = 3 per group; One-way ANOVA with Holm–Sidak's multiple comparisons test; * *p* < 0.05, ** *p* < 0.01, *** *p* < 0.001). **c** Representative microphotographs of rRNA and NeuN immunofluorescence stainings in brain tissue cyrosections of mice subjected either to ischemic stroke or sham surgery: rRNA (red), NeuN (green) and nuclei (blue). Scale bars = 30 µm and 15 µm (magnified sections)

As compared to sham-operated animals, the amount of nuclear, peri-nuclear as well as non-nuclear rRNA in neurons was significantly increased at 2 h and 4 h of reperfusion across the entire ischemic ipsilateral hemisphere of stroke-subjected mice (Fig. 4a, c). A more detailed analysis revealed that within the ipsilateral striatum, representing the infarct core, the abundance of neuronal rRNA, localized distant from the nucleus, was significantly enhanced after 2 h and 4 h of reperfusion (Fig. 4a). Similarly, in the ipsilateral cortex, representing the ischemic peri-infarct area, the amount of neuron-derived nuclear, peri-nuclear and non-nuclear rRNA was significantly increased at 2 h and 4 h of reperfusion in comparison to sham surgery controls (Fig. 4a). The quantity of non-nuclear but not nuclear or peri-nuclear rRNA from non-neuronal cells was significantly increased across the entire ipsilateral hemisphere of stroke-subjected animals after 2 h and 4 h of reperfusion, albeit to a lesser extent compared to neurons (Additional file 4: Fig. S4). Within the ipsilateral striatum and cortex of stroke-subjected mice, the amount of non-nuclear rRNA originating from non-neuronal cells was non-significantly increased as compared to sham-operated animals (Additional file 4: Fig. S4). By contrast, in both neurons and non-neuronal cells localized within the entire contralateral brain hemisphere, including cortex and striatum of mice subjected to ischemic stroke, the abundance of nuclear, peri-nuclear and non-nuclear rRNA was not significantly different in comparison to sham-operated mice (Fig. 4a, c; Additional file 4: Fig. S4). In addition, the increase in non-nuclear rRNA was accompanied by a significant transcriptional up-regulation of various pro-inflammatory factors in the ischemic ipsilateral hemisphere as compared to sham-operated animals (Fig. 4b).

**Fig. 5:**
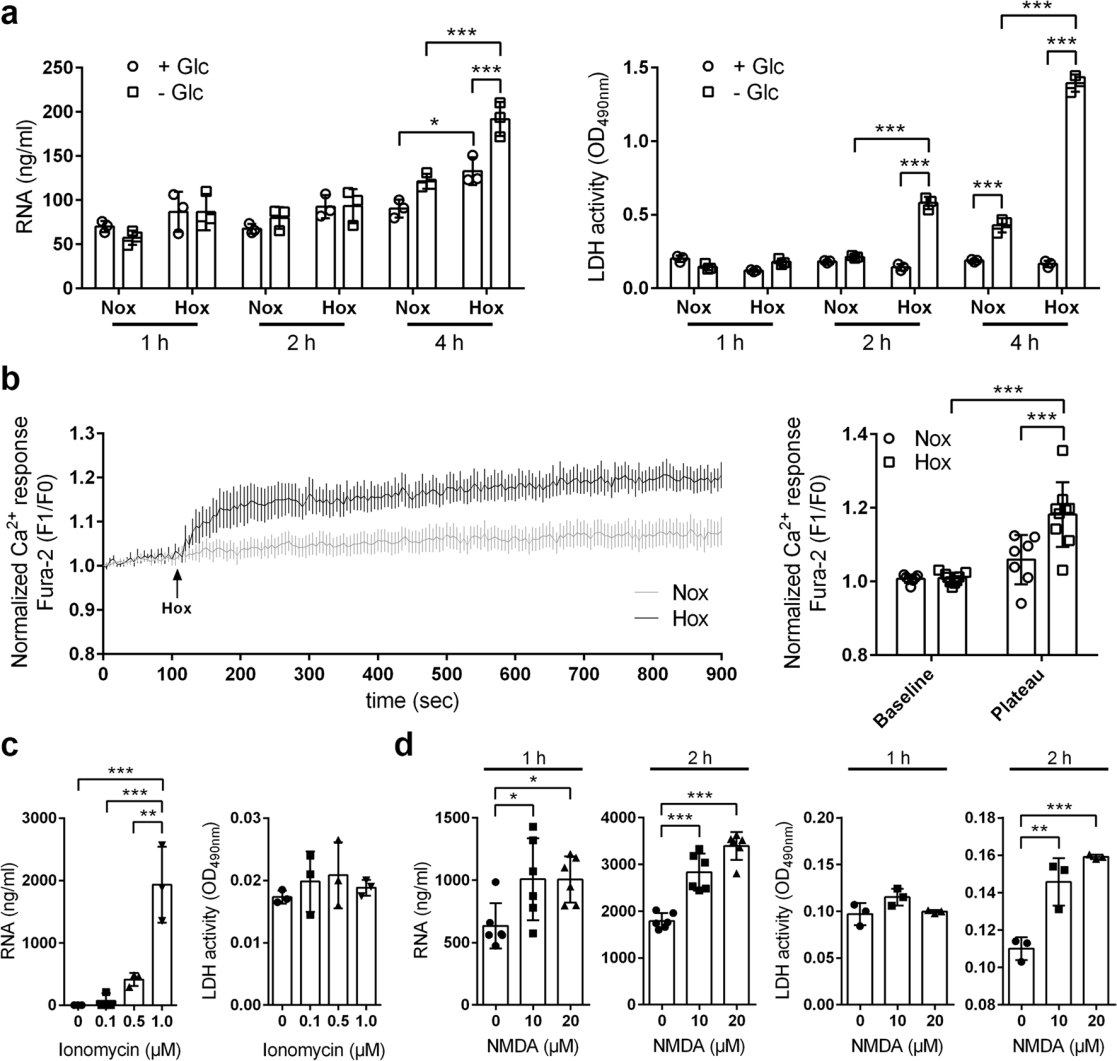
Hypoxia/ischemia and cytosolic calcium rises trigger neuronal RNA release. **a** Neuronal HT-22 cells were incubated in glucose-containing (+ Glc) or glucose-free (-Glc) medium under normoxic (Nox) or hypoxic (Hox; 1% O_2_) conditions for 1, 2 or 4 h. The eRNA levels in cellular supernatants were quantified by ELISA. Cell viability was determined by measuring extracellular LDH activity (*n* = 3 per group; Two-way ANOVA with Holm–Sidak's multiple comparisons test; * *p* < 0.05, ** *p* < 0.01, *** *p* < 0.001). **b** Cytoplasmic free calcium concentration in HT-22 cells exposed to normoxic or hypoxic conditions were determined using the ratiometric Ca^2+^ indicator dye Fura-2 AM (*n* = 7–9 per group; Two-way ANOVA with Holm–Sidak's multiple comparisons test; * *p* < 0.05, ** *p* < 0.01, *** *p* < 0.001). The normalized Ca^2+^ response is expressed as F1/F0 ratio (F1, Fura-2 340/380 ratio at indicated time point; F0, Fura-2 340/380 ratio at baseline). **c** Neuronal HT-22 cells were treated with 0.1, 0.5 or 1 µM ionomycin for 1 h. Untreated cells served as control. The eRNA levels in cellular supernatants were quantified by ELISA. Cell viability was determined by measuring extracellular LDH activity (*n* = 3 per group; One-way ANOVA with Holm–Sidak's multiple comparisons test; * *p* < 0.05, ** *p* < 0.01, *** *p* < 0.001). **d** Primary cortical neurons were treated with 10 or 20 µM NMDA in the presence of 10 µM glycine for 1 or 2 h. Cells treated with 10 µM glycine only were used as control. The eRNA concentration in cellular supernatants was quantified by ELISA. Cell viability was determined by measuring extracellular LDH activity (*n* = 3–6 per group; One-way ANOVA with Holm–Sidak's multiple comparisons test; *p < 0.05, ** *p* < 0.01, *** *p* < 0.001)

We next investigated if oxygen, glucose or combined oxygen–glucose deprivation (OGD) would trigger RNA release from neurons. The amount of eRNA in cultured neuronal HT-22 cells exposed to either oxygen or combined oxygen–glucose deprivation for 4 h was significantly increased as compared to normoxic controls (Fig. 5a). Cell viability was significantly reduced upon 2 h and 4 h OGD, but remained unaffected during hypoxic conditions (Fig. 5a), indicating that RNA release upon ischemic stress mainly occurs in a passive manner by necrotic cell death, whereas pure hypoxia triggers active secretion of intracellular RNA without increasing extracellular RNase activity (Additional file 1: Fig. S5a). The RNA release upon hypoxic exposure was accompanied by a rise of cytosolic calcium (Fig. 5b). Accordingly, short-term treatment with the calcium–ionophore ionomycin increased the abundance of eRNA in a concentration-dependent manner (Fig. 5c) without affecting neuronal viability (Fig. 5c) and extracellular RNase activity (Additional file 1: Fig. S5b).

Furthermore, primary murine neurons that express functional glutamate receptors responded to selective NMDA receptor stimulation (which triggers calcium influx) with eRNA release (Fig. 5d). The release of eRNA upon NMDA treatment for 1 h was not accompanied by decreased cell viability, pointing to a predominantly active secretion of intracellular RNA (Fig. 5d). During prolonged treatment with NMDA for 2 h, however, neuronal viability was substantially impaired, which is in accordance with the concept of NMDA receptor-triggered glutamate excitotoxicity (Fig. 5d).

## Discussion

Astrocytes, besides microglia, are the most abundant glia and immune cells in the CNS but their role in innate immunity remains poorly understood. Astrocytes fulfil homeostatic regulatory functions and can become activated in pathological situations such as infection, brain injury, or neurodegenerative diseases [23, 24]. During immunological reactions, astrocytes mainly contribute with an inflammatory response, whereas the role of microglia is more directed towards phagocytic activity [25].

Astrocytes are known to express several TLRs [26], but their mode of activation and their contribution under inflammatory or pathological conditions in the brain remains unclear. Here, we show that extracellular RNA (eRNA), released under a variety of stress conditions such as hypoxia/ischemia, strongly activates astrocytes in the presence of the TLR2 ligands Pam2CSK4 and HMGB1 in a synergistic manner. The combined stimulatory action of eRNA together with Pam2CSK4 or HMGB1 resulted in a significant expression and release of pro-inflammatory cytokines.

In principle, the activation of TLR2 results in the activation of the Myd88-dependent pathway to induce IκB phosphorylation in a stimulated cell. Phosphorylated IκB then dissociates from the complex and activates the transcription factor NF-κB and MAPK signaling pathways, which in turn activate the transcription factor activator protein-1 (AP-1). Activation and translocation of NF-κB and AP-1 into the nucleus then contribute to the expression of pro-inflammatory cytokines [27]. We demonstrate that the eRNA/Pam2CSK4-induced mRNA expression of TNF-α and Il-6 involves the activation of these pathways, whereby only the expression of IL-6 was independent on the activation of the extracellular-signal-regulated kinase (ERK) pathway.

Together, these results clearly reveal that self-eRNA acts in synergy with exogenous danger signals to promote an inflammatory phenotype in astrocytes, which is in accordance with our previous observations in macrophages [14]. In line with our results, it was previously demonstrated that following lipopolysaccharide-mediated TLR2/4 activation in astrocytes, TNF-α or IL-1β self-regulated and modulated the expression of pro-inflammatory cytokines and chemokines [28]. All these findings support the role of astrocytes in innate immune responses and their involvement in CNS pathologies.

Our study further demonstrates that self-eRNA not only acts synergistically with PAMPs but also with DAMPs, which serve as body’s own alarm signals under, e.g., hypoxia/ischemia. In fact, such sterile inflammatory processes contribute to various neurological disorders, including ischemic stroke. Various DAMPs are recognized by TLRs and thus induce an inflammatory response [29]. HMGB1, a non-histone DNA-binding protein, has recently emerged as an extracellular signaling factor to serve as a “necrotic marker” and activator of the innate immune system, leading to inflammatory reactions by utilizing different receptors such as TLR2, TLR4 or receptor for advanced glycation end products (RAGE) [30]. Accordingly, inhibition of HMGB1 transport from the nucleus to the cytoplasm reduced cell injury by regulation of the TLR4/NF-κB pathways and thereby decreased the levels of inflammatory factors [31]. Directly after an ischemic insult, HMGB1 was shown to be massively released into the extracellular space and to subsequently induce neuroinflammation in the post-ischemic brain, thus providing evidence that HMGB1 acts as a mediator that links ischemia-induced acute cell damage and subsequent inflammatory processes [32]. Our study now demonstrates that eRNA is released by neurons during acute cell stress or damage, and accelerates HMGB1-induced pro-inflammatory activity in astrocytes, involving the activation of TLR2. Thus, eRNA decreases the threshold of astrocytic inflammatory activity for PAMPs and DAMPs (Pam2CSK4 and HMGB1). It remains to be investigated whether eRNA also synergizes with other DAMPs or PAMPs for promoting its inflammatory activity.

We have previously demonstrated in a rat stroke model that eRNA can aggravate ischemic injury by inducing vascular permeability via vascular endothelial growth factor (VEGF) [7, 33], and that pre-treatment of animals with RNase led to vessel protection accompanied by decreased edema formation as well as reduced infarct volume [11]. Thus, small amounts of eRNA can act as a requisite cofactor to engage the binding of ligands such as VEGF or cytokines to their cognate receptors.

Mast cells, which contribute to inflammatory reactions during cerebral I/R injury [34] might also be involved in the release of eRNA. Our previous results demonstrated that mast cells during their degranulation process can liberate high amounts of microvesicle-associated eRNA, which induced the expression and release of cytokines from endothelial cells [35]. The release of eRNA from mast cells is an active process and dependent on an increase of intracellular Ca^2+^ [35].

As demonstrated in this study, increased intracellular Ca^2+^ may also contribute to the release of eRNA from neurons upon challenge by sub-lethal hypoxia. Accordingly, treatment of neurons with nontoxic doses of ionomycin or NMDA, drugs that facilitate substantial Ca^2+^ influx, enhanced the release of eRNA as well. Hypoxia/ischemia leads to ATP depletion with subsequent cell damage and massive neuronal release of glutamate finally leading to Ca^2+^-dependent excitotoxic neuronal cell death [36, 37]. Here, we show that neuronal cell death induced by either prolonged ischemic exposure or over-activation of NMDA-type glutamate receptors is associated with a strong liberation of eRNA.

Together, our data strongly indicate that active and passive cellular release of eRNA might have a central role during cerebral ischemia. Indeed, we here show in mice subjected to acute ischemic stroke that the amount of non-nuclear eRNA, presumably representing rRNA released by neurons and other resident brain cells into the extracellular space, was elevated along the ipsilateral striatum and cortex, encompassing infarct core and peri-infarct area, respectively. Moreover, the gain of non-nuclear eRNA early during cerebral I/R injury was accompanied by a gradual up-regulation of pro-inflammatory factors in the ipsilateral brain hemisphere. This would indicate that eRNA released from functionally compromised and dying neurons may contribute to neuroinflammation during cerebral I/R injury as well as other neurological diseases by amplifying inflammatory reactions induced by DAMPs or PAMPs.

## Conclusions

The present results demonstrate that eRNA release is increased during cerebral ischemia and sensitizes astrocytes towards exogenous and endogenous activators of inflammation (PAMPs and DAMPs) in a synergistic manner. These findings indicate a prominent augmenting role for eRNA in the pathogenesis of ischemic stroke and potentially other neurological disorders, and may point towards administration of RNase as an effective treatment against inflammatory brain pathologies.

## Supplementary Information

**Additional file 1: Fig. S1.** Pro-inflammatory activation of astrocytes upon stimulation with self-extracellular RNA/Pam2CSK4 complex is disrupted by RNase pre-treatment. 1 µg/µl RNA was incubated in the absence or presence of 1 ng/µl RNase for 1 h at 37 °C prior to incubation with 100 pg/µl Pam2CSK4 for 2 h at 37 °C. Astrocytes were treated for 8 h with Pam2CSK4/eRNA (± RNase pre-treatment) molecule complexes. Untreated cells were used as control. Real-time RT-PCR was used to determine transcript levels of pro-inflammatory cytokines. Values are normalized to *Rps12* (*n* = 3 per group; One-way ANOVA with Holm–Sidak's multiple comparisons test; * *p* < 0.05, ** *p* < 0.01, *** *p* < 0.001).

**Additional file 2: Fig. S2:** Self-extracellular RNA/Pam2CSK4 complex promotes toll-like receptor expression in astrocytes. (a) Astrocytes were treated for 8 h with either 100 pg/ml Pam2CSK4, 1 µg/ml eRNA or Pam2CSK4/eRNA molecule complexes, prepared by incubation of 100 pg/µl Pam2CSK4 and 1 µg/µl RNA for 2 h at 37 °C. (b–d) Astrocytes were treated for 4 h with either (b) 1 µg/ml MAb-mTLR2 **(c)** 5 µM Bay 11–7084, **(d)** 20 µM PD98059 or 10 µM SB203580 prior to stimulation with Pam2CSK4/eRNA molecule complexes for 8 h. Real-time RT-PCR was used to determine transcript levels of toll-like receptor genes. Values are normalized to *Rps12* (*n* = 3–4 per group; One-way ANOVA with Holm–Sidak's multiple comparisons test; * *p* < 0.05, ** *p* < 0.01, *** *p* < 0.001).

**Additional file 3: Fig. S3:** Self-extracellular RNA and HMGB1 enhance toll-like receptor 2 expression in astrocytes. Astrocytes were treated for 8 h with 1 µg/ml eRNA and 500 ng/ml rhHMGB1 in the presence or absence of 1 µg/ml MAb-mTLR2. Untreated cells were used as control. Real-time RT-PCR was used to determine transcript levels of toll-like receptor genes. Values are normalized to *Rps12* (*n* = 4 per group; One-way ANOVA with Holm–Sidak's multiple comparisons test; * *p* < 0.05, ** *p* < 0.01, *** *p* < 0.001).

**Additional file 4: Fig. S4. **Acute cerebral I/R injury increases the non-nuclear abundance of rRNA derived from non-neuronal brain cells. Mice underwent 60 min MCAO followed by 2 h or 4 h of reperfusion or were subjected to sham surgery. Co-immunofluorescent staining was performed to determine the nuclear, peri-nuclear and non-nuclear abundance of rRNA in NeuN^−^ cells across the striatum and cortex of the contra- and ipsilateral brain hemisphere (*n* = 3–4 per group; One-way ANOVA with Holm–Sidak's multiple comparisons test; * *p* < 0.05, ** *p* < 0.01, *** *p* < 0.001).

**Additional file 5: Fig. S5.** Effects of hypoxia/ischemia and ionomycin on cell viability and extracellular RNase activity of neurons. (a) RNase activity in cell supernatants of neuronal HT-22 cells incubated in glucose-containing (+ Glc) or glucose-free (-Glc) medium under normoxic (Nox) or hypoxic (Hox; 1% O_2_) conditions for 1, 2 or 4 h (*n* = 3 per group; Two-way ANOVA with Holm–Sidak's multiple comparisons test; * *p* < 0.05, ** *p* < 0.01, *** *p* < 0.001). **(b)** Extracellular RNase activity of neuronal HT-22 cell cultures treated with 0.1, 0.5 or 1 µM ionomycin for 1 h (*n* = 3 per group; One-way ANOVA with Holm–Sidak's multiple comparisons test; * *p* < 0.05, ** *p* < 0.01, *** *p* < 0.001).

## Data Availability

All data generated or analyzed during this study are included in this published article and its additional information file.

## Abbreviations

AP-1: Activator protein-1
CNS: Central nervous system
DAMP: Damage-associated molecular patterns
DIV: Days in vitro
ERK: Extracellular-signal-regulated kinase
eRNA: Extracellular RNA
HMGB1: High mobility group box 1
IL-1β: Interleukin-1 beta
IL-6: Interleukin-6
MAPK: Mitogen-activated protein kinase
MCAO: Middle cerebral artery occlusion
MCP-1: Monocyte chemoattractant protein-1
NF-κB: Nuclear factor-kappa B
NMDA: *N*-methyl-d-aspartic acid
OGD: Oxygen–glucose deprivation
PAMP: Pathogen-associated molecular patterns
PRR: Pattern recognition receptor
RAGE: Receptor for advanced glycation end products
ROI: Region of interest
RPS12: Ribosomal protein S12
rRNA: Ribosomal RNA
TLR: Toll-like receptor
TNF-α: Tumor necrosis factor alpha
VEGF: Vascular endothelial growth factor
