# Supplementary figures and images for: Self-extracellular RNA promotes pro-inflammatory response of astrocytes to exogenous and endogenous danger signals
Source: J Neuroinflammation. 2021 Nov 2;18:252. doi: 10.1186/s12974-021-02286-w (PMC8561902; doi:10.1186/s12974-021-02286-w)

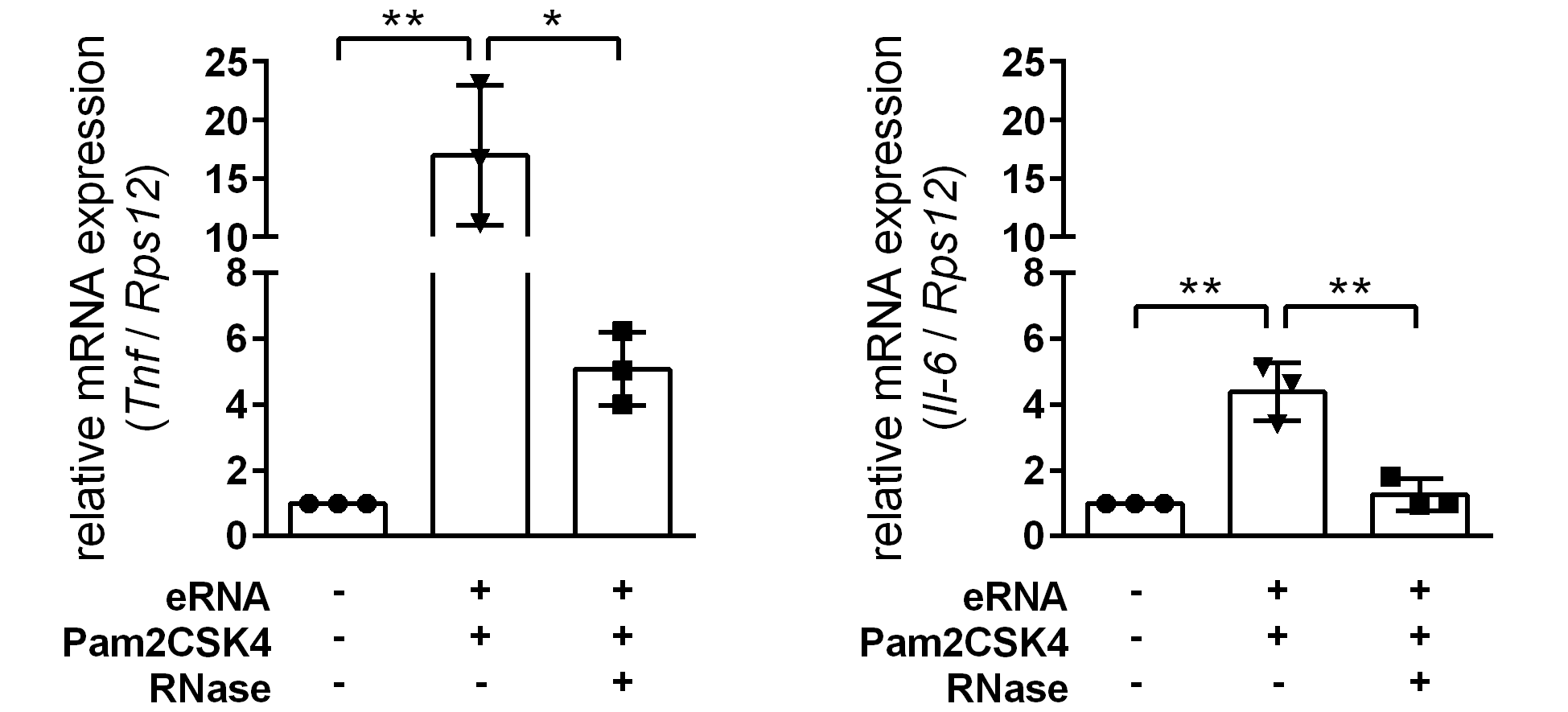

Supplement: Supplementary file 1 — Additional file 1: Fig. S1. Pro-inflammatory activation of astrocytes upon stimulation with self-extracellular RNA/Pam2CSK4 complex is disrupted by RNase pre-treatment. 1 µg/µl RNA was incubated in the absence or presence of 1 ng/µl RNase for 1 h at 37 °C prior to incubation with 100 pg/µl Pam2CSK4 for 2 h at 37 °C. Astrocytes were treated for 8 h with Pam2CSK4/eRNA (± RNase pre-treatment) molecule complexes. Untreated cells were used as control. Real-time RT-PCR was used to determine transcript levels of pro-inflammatory cytokines. Values are normalized to Rps12 (n = 3 per group; One-way ANOVA with Holm–Sidak's multiple comparisons test; * p < 0.05, ** p < 0.01, *** p < 0.001). [file 12974_2021_2286_MOESM1_ESM.tif]

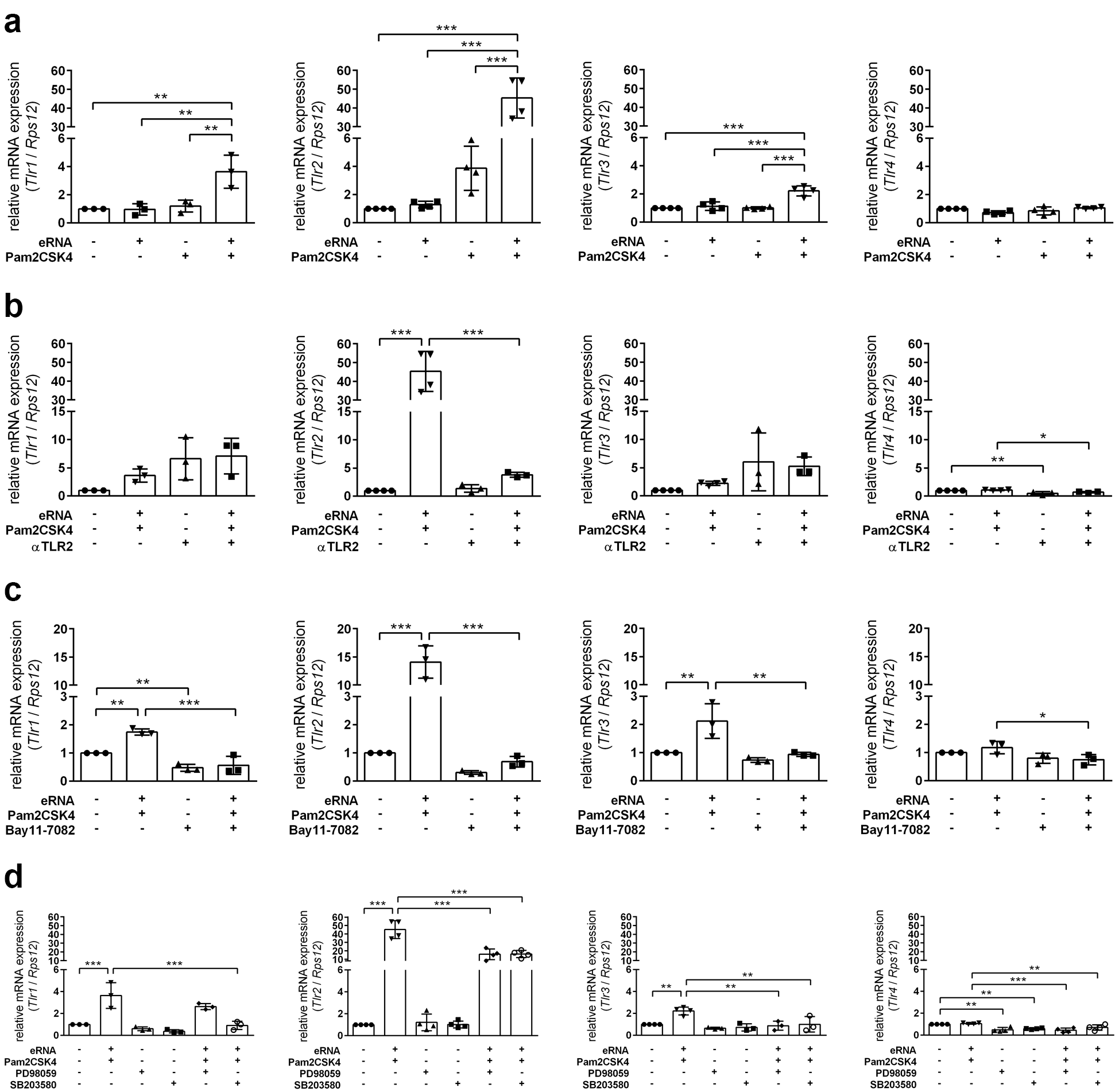

Supplement: Supplementary file 2 — Additional file 2: Fig. S2: Self-extracellular RNA/Pam2CSK4 complex promotes toll-like receptor expression in astrocytes. (a) Astrocytes were treated for 8 h with either 100 pg/ml Pam2CSK4, 1 µg/ml eRNA or Pam2CSK4/eRNA molecule complexes, prepared by incubation of 100 pg/µl Pam2CSK4 and 1 µg/µl RNA for 2 h at 37 °C. (b–d) Astrocytes were treated for 4 h with either (b) 1 µg/ml MAb-mTLR2 (c) 5 µM Bay 11–7084, (d) 20 µM PD98059 or 10 µM SB203580 prior to stimulation with Pam2CSK4/eRNA molecule complexes for 8 h. Real-time RT-PCR was used to determine transcript levels of toll-like receptor genes. Values are normalized to Rps12 (n = 3–4 per group; One-way ANOVA with Holm–Sidak's multiple comparisons test; * p < 0.05, ** p < 0.01, *** p < 0.001). [file 12974_2021_2286_MOESM2_ESM.tif]

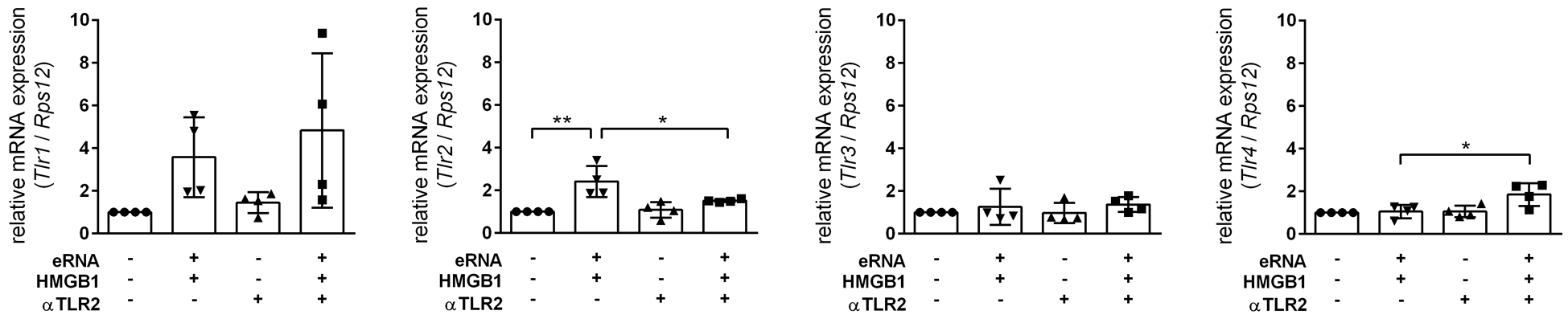

Supplement: Supplementary file 3 — Additional file 3: Fig. S3: Self-extracellular RNA and HMGB1 enhance toll-like receptor 2 expression in astrocytes. Astrocytes were treated for 8 h with 1 µg/ml eRNA and 500 ng/ml rhHMGB1 in the presence or absence of 1 µg/ml MAb-mTLR2. Untreated cells were used as control. Real-time RT-PCR was used to determine transcript levels of toll-like receptor genes. Values are normalized to Rps12 (n = 4 per group; One-way ANOVA with Holm–Sidak's multiple comparisons test; * p < 0.05, ** p < 0.01, *** p < 0.001). [file 12974_2021_2286_MOESM3_ESM.tif]

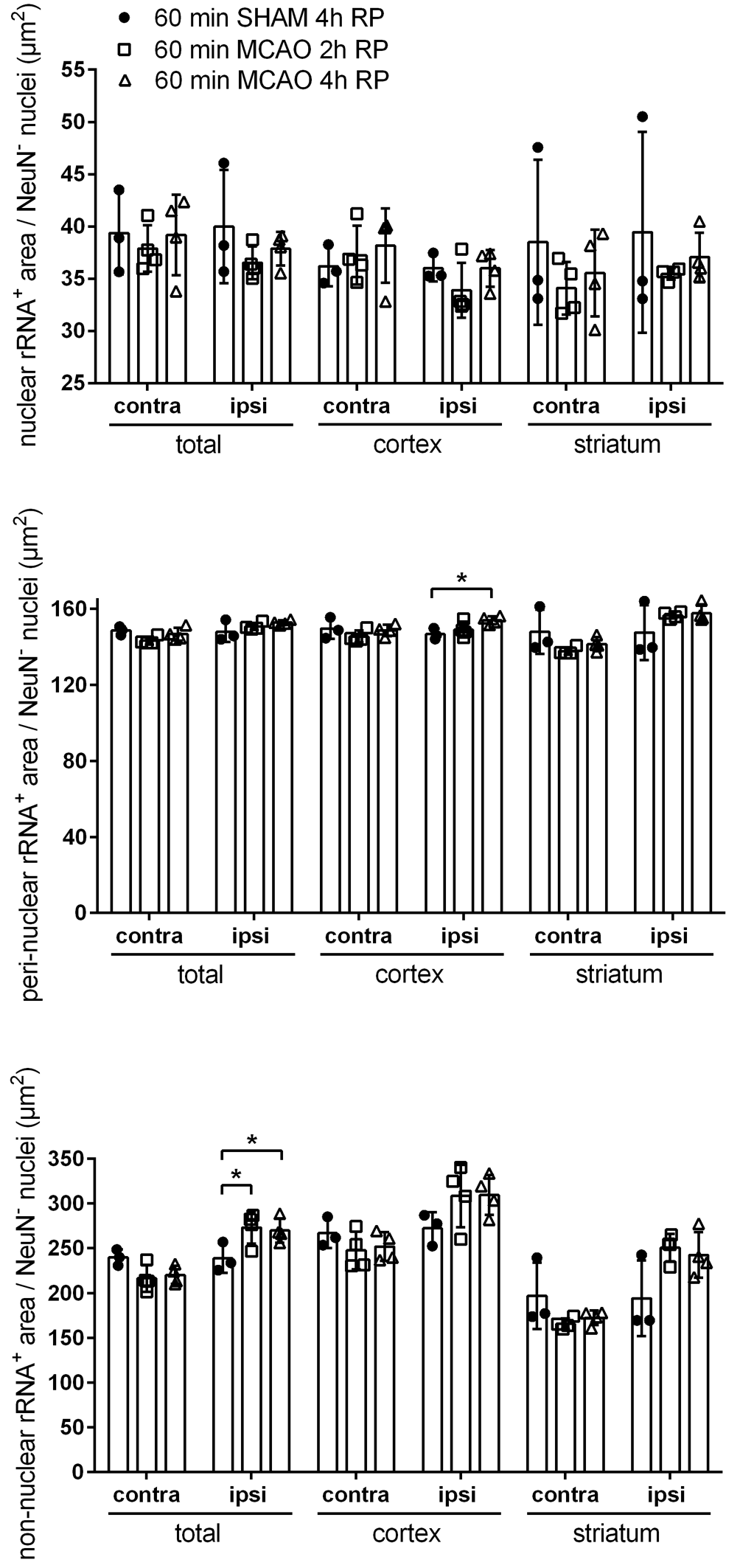

Supplement: Supplementary file 4 — Additional file 4: Fig. S4. Acute cerebral I/R injury increases the non-nuclear abundance of rRNA derived from non-neuronal brain cells. Mice underwent 60 min MCAO followed by 2 h or 4 h of reperfusion or were subjected to sham surgery. Co-immunofluorescent staining was performed to determine the nuclear, peri-nuclear and non-nuclear abundance of rRNA in NeuN− cells across the striatum and cortex of the contra- and ipsilateral brain hemisphere (n = 3–4 per group; One-way ANOVA with Holm–Sidak's multiple comparisons test; * p < 0.05, ** p < 0.01, *** p < 0.001). [file 12974_2021_2286_MOESM4_ESM.tif]

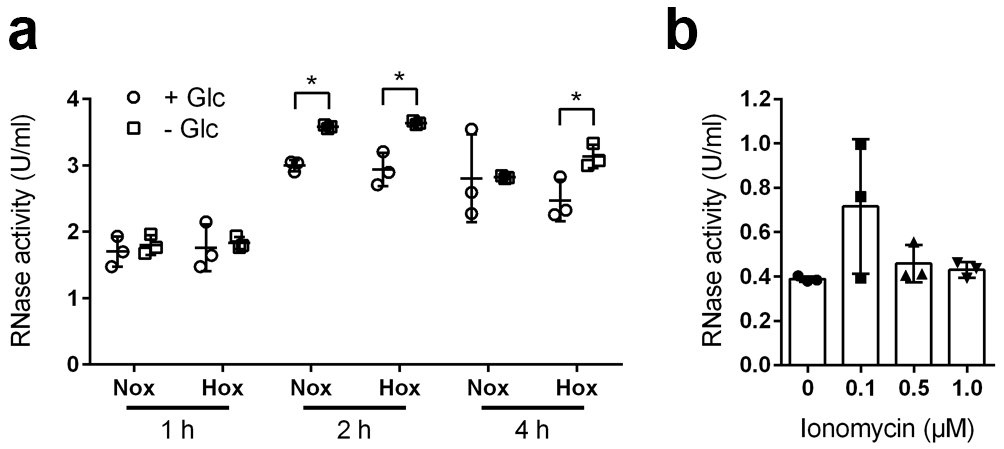

Supplement: Supplementary file 5 — Additional file 5: Fig. S5. Effects of hypoxia/ischemia and ionomycin on cell viability and extracellular RNase activity of neurons. (a) RNase activity in cell supernatants of neuronal HT-22 cells incubated in glucose-containing (+ Glc) or glucose-free (-Glc) medium under normoxic (Nox) or hypoxic (Hox; 1% O2) conditions for 1, 2 or 4 h (n = 3 per group; Two-way ANOVA with Holm–Sidak's multiple comparisons test; * p < 0.05, ** p < 0.01, *** p < 0.001). (b) Extracellular RNase activity of neuronal HT-22 cell cultures treated with 0.1, 0.5 or 1 µM ionomycin for 1 h (n = 3 per group; One-way ANOVA with Holm–Sidak's multiple comparisons test; * p < 0.05, ** p < 0.01, *** p < 0.001). [file 12974_2021_2286_MOESM5_ESM.tif]
